# Overlooked Fracture of the Inferior Scapular Angle Treated Conservatively

**DOI:** 10.1155/2019/9640301

**Published:** 2019-01-10

**Authors:** Kiyohisa Ogawa, Wataru Inokuchi, Takayuki Honma

**Affiliations:** Department of Orthopedic Surgery, Eiju General Hospital, 2-3-23 Higashiueno, Taito-ku, Tokyo 110-8645, Japan

## Abstract

Isolated fracture of the inferior scapular angle is extremely rare. We present the case of a 20-year-old female with persistent periscapular pain and a winged scapula caused by delayed union of an inferior scapular angle (ISA) fracture. Ten months previously, the patient had a car accident while seated in the left rear passenger seat. The patient visited an orthopedic clinic where a surgeon diagnosed left shoulder contusion without any abnormal radiographic findings. The left arm was kept in a sling for 2 months, as left arm elevation caused severe pain in the upper back. After sling removal, the dull pain around the left scapula continued. The patient presented at our clinic because her mother had noticed the deformity of her back. Radiographs showed a small bony fragment in the ventral side of the ISA. Computed tomography revealed a narrow gap between the ISA and the fragment. The patient's symptoms resolved with conservative treatment that consisted of relative rest for 2 months and subsequent reinforcement exercises of the serratus anterior for 2 months.

## 1. Introduction

Fractures of the scapula are relatively rare, constituting only 0.4–0.9% of all fractures and about 3–5% of all fractures of the shoulder girdle [1]. The majority of scapular fractures are the result of high-energy trauma, while low-energy scapular fractures are quite uncommon [2]. Although avulsion fracture is representative of the type of fractures caused by low-energy trauma, avulsion fractures of the scapula resulting from indirect trauma are extremely rare [3, 4]. We present the case of a 20-year-old female with persistent periscapular pain and a winged scapula caused by delayed union of an inferior scapular angle (ISA) fracture most likely avulsed by the serratus anterior (SA) muscle. The patient's symptoms resolved with conservative treatment that consisted of relative rest and reinforcement exercise of the SA. Written informed consent was obtained from the patient for publication of this case report and accompanying images.

## 2. Case Report

A 20-year-old right-hand-dominant and otherwise healthy female student presented with protrusion of the left upper back and left periscapular pain that occurred after sport activities. Ten months previously, the patient had been seated in the left rear passenger seat in a car that was hit in the left side by another car. Further details such as the posture and the arm position of the patient at the time of the accident were uncertain. At the time of the car accident, the patient visited an orthopedic clinic where a surgeon diagnosed left shoulder contusion without any abnormal radiographic findings. The left arm was kept in a sling for 2 months, as left arm elevation caused severe pain in the upper back. After sling removal, the patient returned to basketball, which generated continuous dull pain around the left scapula. She presented at our clinic because her mother had noticed the deformity of her back.

**Figure 1:**
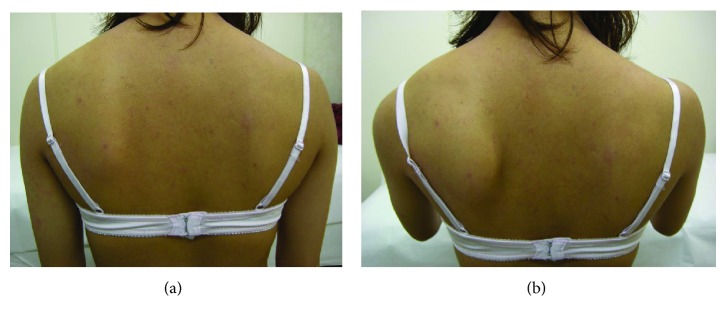
Photographs taken at the time of the first visit. (a) The left scapula was slightly higher than the right scapula and presented with an atypical medial winging with the arm at the side. (b) The winged scapula became prominent during 0–45° of active flexion.

**Figure 2:**
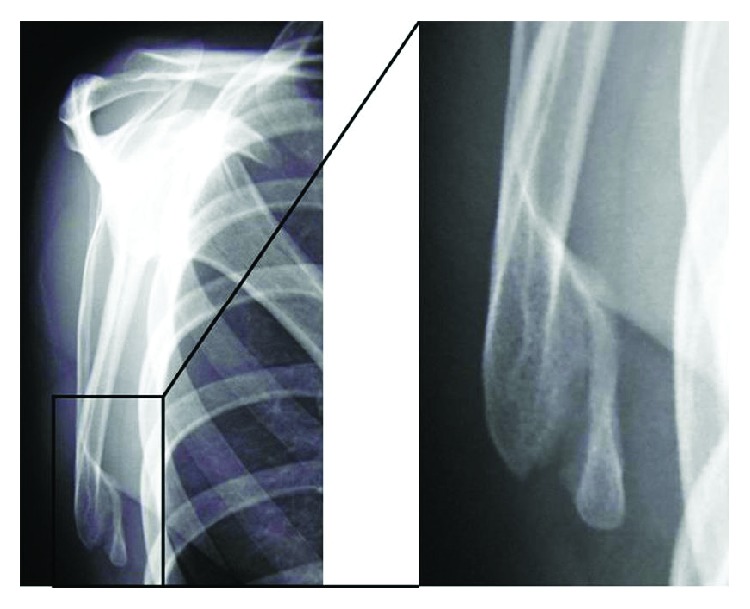
Radiographs showing a small bony fragment on the ventral side of the inferior scapular angle with a narrow space between the fragment and the scapular body to which the superior border was connected by a callus bridge.

**Figure 3:**
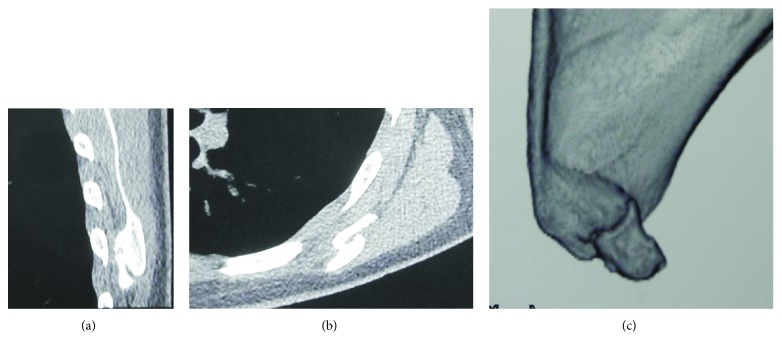
Computed tomography at the time of the first visit. (a) Sagittal section revealing that the callus did not connect the fragment with the scapular body. (b) Axial section demonstrating the lateral displacement of the fracture fragment. (c) Three-dimensional reconstruction image showing a bony protrusion ranging from the medial scapular border to the bony fragment, with a narrow break between the protrusion and fragment.

The patient had no relevant family or medical history. There was no neurological deficit in the left shoulder and arm. The left scapula was slightly higher than the contralateral scapula and exhibited atypical medial winging with the arm at the side. The distance between the spinal process and medial scapular border was shorter on the left side than the right side at the inferior angle level, but these distances were almost the same at the scapular spine level (Figure 1(a)). Contraction of the scapular stabilizing muscles was good. There was a palpable bony protuberance without tenderness on the ventral side of the ISA. The limitations of the active ranges of motion of the left shoulder compared with the right shoulder were 25° for total elevation, 15° for external rotation, and none for internal rotation and horizontal adduction; however, there were no limitations of the passive ranges of motion. The winged scapula became prominent at 0–45° of active flexion, while it disappeared when the patient flexed the left arm while consciously attempting to depress the scapula (Figure 1(b)). The winged scapula did not emerge when the patient pushed on a wall at chest level. Radiographs showed a small bony fragment in the ventral side of the ISA, with a narrow space between the fragment and the scapular body (Figure 2). Computed tomography revealed a bony protrusion extending from the medial scapular border to the bony fragment, with a narrow gap between the protrusion and the fragment (Figures 3(a)–3(c)).

**Figure 4:**
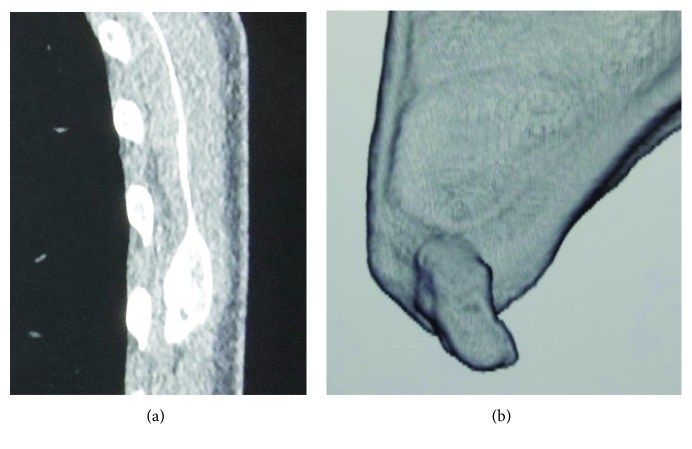
Computed tomography at the time of the second visit 4 months later. (a) Sagittal section revealing the disappearance of the narrow space between the fragment and the scapular body. (b) Three-dimensional reconstruction image showing the disappearance of the narrow break between the protrusion and the fragment.

The patient was instructed to avoid elevating the left arm for 2 months and then performed reinforcement exercises of the SA such as the scapular push-up and the bear hug using an elastic band for 2 months. At examination 4 months later, the periscapular pain and the winging of the scapula with the arm at the side and in active flexion had resolved. The push-on-the-wall test at waist level was negative, and the range of motion of the left arm was the same as the unaffected side, except for a 15° limitation in external rotation. Although the radiographic findings were the same as at the first visit, computed tomography demonstrated bony union (Figures 4(a) and 4(b)). The patient was permitted to use the left arm without restrictions.

At the time of the final follow-up 10 years of postinjury, the patient reported that there was an occasional painless click and a sporadic floating feeling of the scapula with initial active flexion of the arm. However, there was no pain or any disturbance to the patient's activities of daily life and work as a physical therapist. The patient's colleague confirmed the disappearance of the winged scapula associated with shoulder movement. The DASH score was 0, and the Constant score ratio compared with the right shoulder was 100% [5, 6].

## 3. Discussion

As the scapula is protected from direct forces by skeletal muscles and moves almost freely on the flexible chest wall, fractures of the scapula are relatively rare. The majority of scapular fractures are the result of high-energy trauma, while low-energy scapular fractures are quite uncommon [2]. Avulsion fracture is representative of the fractures caused by low-energy trauma. Avulsion fractures of the scapula resulting from indirect trauma, such as the pull exerted by muscles or ligaments on their bony insertion, are extremely rare [4], representing 0.01% of all skeletal fractures and 2% of scapular fractures [3]. There are reportedly three mechanisms by which scapular avulsion fractures may occur: (1) uncoordinated muscle contraction due to electroconvulsive therapy, electric shocks, or, more rarely, epileptic seizures with the presence of abnormal bone [7], (2) resisted muscle pull because of trauma or unusual exertion, and (3) avulsion of a ligamentous attachment [8]. Stress fractures at muscle attachments are another type of fracture caused by low-energy trauma and may occur due to repeated trauma to the bone or repetitive muscular contraction [8]. Stress fractures of the scapula, both the fatigue type (abnormal stress or torque on a bone with normal elastic resistance) and the insufficiency type (normal stress on a bone with deficient elastic resistance), have been described in various patient populations and anatomical locations [9, 10].

The existence of ISA fracture has been recognized since 1798 [11, 12]. To the best of our knowledge, there have been 14 cases of ISA fracture previously reported in English that were radiographically verified or included detailed descriptions of the fracture condition (Table 1). The patients' ages range from 13 to 70 years, indicating that ISA fracture may occur in any age. The ISA develops from the secondary ossification center, which appears at around 15–17 years of age and generally fuses by 23 years [13, 14]; hence, the possibility of epiphysiolysis cannot be excluded completely in four of the cases in which the patients were aged 20 years or less, including our case [8, 15, 16].

In most previous reports, ISA fracture was considered to be an avulsion fracture caused by the pull of the SA [8, 15, 17, 18]. However, diagnosis of avulsion fractures is based on the following vague criteria: (a) the absence of a history of violent direct trauma and (b) the anatomical location of the fractures in relation to scapular muscle attachments [8]. Hence, definitive diagnosis of avulsion fracture is often difficult or questionable. In the seven previously reported cases of ISA fracture in which the details at the time of the injury were clearly described, two cases had experienced an impact to the ISA [19, 20] and one chronic case had the scar of a contused wound on the ISA [21]. A direct impact was denied in the other four cases [8, 15, 22, 23], of which two cases had bone insufficiency: one was an insufficiency type stress fracture related to coughing [22] and the other was properly classified as an insufficiency fracture caused by convulsion [23]. Therefore, the mechanism of ISA fracture varies and it is questionable to indiscriminately consider it as an avulsion fracture. Even though the present patient could not recall the details of the accident, we consider that it was probably an avulsion fracture, as the patient's mother had not noticed any signs of bruising (such as subcutaneous bleeding or contusions) on the patient's back on the day of the injury.

The fractured fragment is displaced by the traction of the attached muscles. When the fractured fragment of the ISA is small, the lower part of the SA and a part of the latissimus dorsi originating from the scapula remain attached to it; when the fragment is large, the lower parts of the rhomboid major and teres major may also be attached to it [17, 19]. The SA consists of digitations arising from the upper eight to ten ribs and intercostal fascia and is divided into three parts: upper, middle, and lower; the lower part is formed by the lower four to five digitations and is attached to the ISA [24, 25]. The powerful lower part of the SA pulls the fracture fragment inferolaterally. However, other attached muscles reduce the inferolateral displacement as they pull superolaterally or superomedially. Given that the direction of the pull of any of the abovementioned muscles corresponds with the ribcage, the fractured fragment never separates from the ribcage. The remaining scapular body relocates superiorly, while its inferior part moves medially and separates from the ribcage because of the loss of the inferolateral traction of the powerful lower part of the SA; this causes the atypical medial winged scapula. Consequently, the fractured fragment is always situated on the ventral side of the scapular body. Winged scapula was observed in seven (88%) of eight cases that precisely reported the situation of the scapular body [8, 15, 17, 19–21, 26]. In our case, although the fractured fragment attached to the ventral side of the lowest scapular body, the scapula presented with winging. As there was a trapezoidal callus on the ventral side of the scapular body medial to the fractured fragment, we supposed that a periosteal sleeve was formed at the time of injury and restrained the displacement of the fragment.

The symptoms experienced in the acute phase of ISA fracture are common in most fractures. The general symptoms in the subacute and chronic phase are an inability to elevate the arm above shoulder level, weakness of arm elevation, and periscapular pain with arm elevation [16, 17, 19–21, 26]. However, a full active range of motion was maintained in two previously reported cases [8, 20] (Table 2). Confirmation of the diagnosis of ISA fracture requires demonstration of the fractured fragment on imaging. In radiography, a precise lateral view of the scapula is needed to reveal the fractured fragment, as it is difficult to identify on anteroposterior view due to overlapping with the ribcage. ISA fracture was overlooked at the time of injury in two of 12 previously reported cases in which the clinical histories were appropriately described [20, 21]. In our case, the fracture was overlooked at the previous clinic the patient visited (Table 2). Therefore, as ISA fractures are easily missed on conventional radiography of the shoulder, it is essential to take an accurate lateral view of the scapula. As shown in our case, CT is also useful for the detailed observation of the fracture site and fractured fragment.

Winged scapula is the most common presenting symptom, with an incidence of 88% in ISA fracture cases [8, 15, 17, 19–21, 26]. The causes of winged scapula vary, and the etiology is anatomically classified as type I (nerve), type II (muscle), type III (bone), and type IV (joint) [27]. Of these, winged scapula that does not depend on SA paralysis is defined as pseudowinging [28]. The winged scapula seen in ISA fracture is an extremely specific type of pseudowinging. In our case, the affected scapula was slightly high, and its lower part was displaced medially; this medial displacement became prominent during active flexion of 0-45°, while the upper scapula remained in its normal position with the arm at rest and during active arm flexion. The winged scapula observed in our case differs from the typical medial winging associated with long thoracic nerve palsy. Moreover, as the winging disappeared when the present patient flexed the arm while consciously attempting to depress the scapula, SA paralysis could be excluded. Another potential etiology of winged scapula is avulsion of the SA [17, 29–33]. SA avulsion can be distinguished from ISA fracture by the existence of pressure pain at the origin or insertion of the SA and by MRI findings [17, 30–33]. However, simultaneous ISA fracture and total avulsion of the SA insertion has been reported [17]. When prominent typical medial winging of the scapula cannot be explained by insufficiency of the lower part of the SA due to ISA fracture, associated total avulsion of the SA insertion should be considered.

Regarding treatment of ISA fracture, 6 of 11 reported cases that precisely described the treatment methods were treated surgically [16, 17, 19–21, 26], and five cases underwent conservative treatment [8, 15, 22, 23]. In the surgically treated cases, reduction and fixation of the fracture fragment was performed in four cases, and excision of the fragment was performed in two cases; there were no postoperative physical impediments, except in one patient who underwent reduction and fixation of the fragment [26]. In contrast, there was a slight physical impediment in three of the five cases receiving conservative therapy [8, 22]. In our case, at the time of the first visit to our clinic, bone union was delayed due to the continuation of inappropriate activities in daily life and sporting activity because of the previous wrong diagnosis. Activity of the lower part of the SA was restricted by prolonged pain, which was considered to be the cause of the winged scapula. Moreover, the decrease in active external rotation that persisted even after treatment seems to be a result of a decrease in external rotation strength that may have been indirectly related to a weakness of the scapula stabilizers rather than the external rotators of the shoulder [34]. When the proper physical therapy according to progression of bony union is not performed [15], long-term muscle dysfunction may persist.

## Figures and Tables

**Table 1:** Details of the previously reported cases of inferior scapular angle fracture (part 1).

Case no.	Year	Author(s)	Patient age/sex/affected side	Causes of injury	Direct trauma
1	1924	Longabaugh [26]	26/male/?	Automobile accident	?
2	1954	Kelly [7]	?/?/?	Electroconvulsive therapy	?
3	1975	Imatani [18]	?/?/?	?	?
4	1977	Peraino et al. [23]	57/male/left	Grand mal convulsion	—
5	1981	Hayes and Zehr [17]^17^	25/male/right	?	?
6	1982	Heyse-Moore and Stoker [8]	70/male/right	Fall forwards	—
7			50/male/right	Electric shock, fall backwards	?
8			13/female/left	Tobogganing accident	?
9	1998	Gupta et al. [21]	45/male/left	A pallet of bricks fell on him	Healed laceration
10	1998	Brindle and Coen [15]	17/male/right	Wrestling (arm-bar)	—
11	2002	Kaminsky and Pierce [16]	16/male/right	Football tackle (indirect)	?
12	2004	Franco et al. [22]	47/male/left	DM, hemodialysis, prednisone, prolonged cough	—
13	2010	Mansha et al. [19]	31/male/right	Thrown from a car	+
14	2016	Speigner et al. [20]	51/male/right	Fell down the stairs and directly hit the inferior angle	+
15		Our case	20/female/left	Automobile accident	?

?: unknown; DM: diabetes mellitus.

**Table 2:** Details of the previously reported cases of inferior scapular angle fracture (part 2).

Case no.	Winging	Other symptoms	Duration from injury to final treatment	Treatment	Residual physical impediments
1	Winged outward	Unable to raise the arm above shoulder level	1 month	S	Some weakness
2	?		?	?	?
3	?		?	?	?
4	?		Immediate	C	?
5	+	Weakness, tired easily, grating sensation	10 months (early diagnosed)	S	None
6	?		Immediate	C	10° abduction loss
7	?		Immediate	?	?
8	+	Full movement and power	23 days	C	Clicking
9	Medial ++	Scapular prominence, pain, restricted ROM	7 months (overlooked)	S	None
10	Medial ++	Unable to raise arm	Immediate	C	None
11	—	Persistent pain	3 months (early diagnosis)	S	None
12	?	Mild pain during abduction movement	Immediate	C	Mild pain on abduction
13	Medial ++	Persistent pain, reduced power	2 years (early diagnosis)	S	None
14	Medial ++	Full ROM, persistent weakness	5 months (overlooked)	S	None
15	Medial ++	Full ROM, pain after activities	10 months (overlooked)	C	Occasional clicking

?: unknown; medial: medial winging; ROM: range of motion; S: surgery; C: conservative treatment.
